# Resistance training promotes reduction in Visceral Adiposity without improvements in Cardiomyocyte Contractility and Calcium handling in Obese Rats

**DOI:** 10.7150/ijms.42612

**Published:** 2020-07-11

**Authors:** Alexandre Barroso Melo, Andressa Prata Leite Damiani, Priscila Murucci Coelho, Arícia Leone Evangelista Monteiro de Assis, Breno Valentim Nogueira, Lucas Guimarães Ferreira, Richard Diego Leite, Rogério Faustino Ribeiro Júnior, Ana Paula Lima-Leopoldo, André Soares Leopoldo

**Affiliations:** 1 Centre for Physical Education and Sports, Department of Sports, Federal University of Espírito Santo, Vitória, Espírito Santo, Brazil.; 2 Centre for Health Sciences, Department of Nutrition, Federal University of Espírito Santo, Vitória, Espírito Santo, Brazil.; 3 Center of Health Sciences, Department of Morphology, Federal University of Espírito Santo, Vitória, Espírito Santo, Brazil.; 4 Center of Health Sciences, Department of Physiological Sciences, Federal University of Espírito Santo, Vitória, Espírito Santo, Brazil.

**Keywords:** high-fat diet, obesity, resistance training, contractile function, calcium handling

## Abstract

Resistance training (RT) improves the cardiomyocyte calcium (Ca^2+^) cycling during excitation-contraction coupling. However, the role of RT in cardiomyocyte contractile function associated with Ca^2+^ handling in obesity is unclear. Wistar rats were distributed into four groups: control, sedentary obese, control plus RT, and obesity plus RT. The 10-wk RT protocol was used (4-5 vertical ladder climbs, 60-second interval, 3× a week, 50-100% of maximum load). Metabolic, hormonal, cardiovascular and biochemical parameters were determined. Reduced leptin levels, epididymal, retroperitoneal and visceral fat pads, lower body fat, and adiposity index were observed in RT. Obesity promoted elevation of collagen, but RT did not promote modifications of LV collagen in ObRT. RT induced elevation in maximum rates of contraction and relaxation, and reduction of time to 50% relaxation. ObRT group did not present improvement in the cardiomyocyte contractile function in comparison to Ob group. Reduced cardiac PLB serine^16^ phosphorylation (pPLB Ser^16^) and pPLB Ser^16^/PLB ratio with no alterations in sarcoplasmic reticulum Ca^2+^ ATPase (SERCA2a) and phospholamban (PLB) expression were observed in Ob groups. Resistance training improved body composition reduced fat pads and plasma leptin levels but did not promote positive alterations in cardiomyocyte contractile function, Ca^2+^ handling and phospholamban phosphorylation.

## Introduction

Obesity is a chronic metabolic disease that is characterized by excessive accumulation of adipose tissue 1,2. Currently, it is considered a global epidemic and an important public health issue, besides being an independent risk factor for cardiovascular diseases 3. In this context, a close relationship has been observed between accumulation of adipose tissue and impairment of cardiac function in both human and experimental models 4-9.

Experimental studies have been developed to elucidate the pathophysiological mechanisms related to cardiac functional impairment induced by obesity, including abnormalities in myocardial intracellular calcium (Ca^2+^) handling, one of the major regulatory mechanisms of contractility and relaxation 7,10-12. Thus, impairment of myocardial function assigned to obesity may be related to mechanisms involving the Ca^2+^ influx and release into the cytosol, and/or damage in the recapture and/or extrusion of this ion by the sarcoplasmic reticulum 7,10-13. Generally, relaxation impairment observed in obese rats may be related to phospholamban (PLB) phosphorylation of Ser^16^ and Thr^17^ by protein kinase A (PKA) or CaMKII impairment, provided that both are physiologically relevant to control SERCA2a activity 14,15.

Non-pharmacological approaches, such as physical exercise, have been used as an efficient and viable tool to improve adiposity parameters and minimize and/or reverse the cardiac damage promoted by obesity 16-18. Physical exercise has been able to alter the activity and expression of regulatory proteins, improving contraction and relaxation in several physiological and pathological models 19-22. The literature has consistently shown that endurance training improves myocardial contractility associated with the benefits of intracellular Ca^2+^ handling, as well as promoting increased sensitivity of the myofilaments to this ion in isolated cardiomyocytes 23,24.

Resistance Training (RT) is effective in improving body composition 16,29-31. Other studies also showed that RT was able to reduce the adipocyte area and body fat percentage 16,31. Regarding the role of RT in cardiovascular system, limited data exist about the effects on healthy or unhealthy heart 16,25-28. Nevertheless, it has been demonstrated that RT performed for 1 and 3 months can generate benefits to heart, such as increased LV velocity of contraction and relaxation 25. In addition, RT may have positive effects on cardiac function in cardiopathy patients and experimental models of chronic heart failure and diabetes mellitus 16,26-28. However, these functional benefits observed in the heart depend on the training variables, such as intensity, frequency, duration, and type of exercise used 26.

Nevertheless, the effects of RT on Ca^2+^ handling are poor studied. Melo et al. 19 have** s**hown improvement in contraction and relaxation of cardiomyocytes in healthy rats, which are related to increased protein expression of SERCA2a. However, to the best of our knowledge, no studies evaluated the myocardial Ca^2+^ handling adaptations to chronic RT in obesity. Thus, the aim of this study was to investigate the effects of RT on the process of cardiac remodeling, contractile function and Ca^2+^ handling in obese rats. The hypothesis was that RT promotes improvement in contractile function in obese cardiomyocytes, which may be related to adjustments in Ca^2+^ handling.

## Material and Methods

### Animals and Care

Thirty-day-old male *Wistar* rats (≈ 150 g) obtained from the Animal Quarters of the Federal University of Espírito Santo (Vitória, Espírito Santo, Brazil) were housed in individual cages. The environment was controlled in terms of light (12h light/dark cycle starting at 6 am), clean-air room temperature (23 ± 3°C), and relative humidity (60 ± 5%). All experiments and procedures were conducted in accordance with the “*Guide for the Care and Use of Laboratory Animals” published by* “*U.S. National Institutes of Health*” and current Brazilian laws. The University of Espírito Santo Ethics Committee approved the experimental protocol (CEUA-UFES 16/2016).

### Experimental Design

After 7 days of acclimatization, the rats were distributed into two groups: control (C, n=29), and obese (Ob, n=29). The C group was fed a standard diet (Nuvilab CR1-Nuvital, Colombo, Paraná, Brazil) containing 13.9% of its kcal from fat, 55.9% from carbohydrates, and 30.2% from protein. Ob animals were alternately submitted to four palatable high-fat diets (RC Focus 2413, 2414, 2415, and 2416) containing 49.2% of its kcal from fat, 28.9% from carbohydrates, and 21.9% from protein 7. Each diet was changed daily, and the rats were maintained on their respective diets for 26 consecutive weeks. The high-fat diet was calorically rich (high-fat diet = 3.65 kcal/g versus low-fat diet = 2.92 kcal/g) due to its higher fat energy (consisting of saturated and unsaturated fatty acids, which provided 20% and 80% of the fat-derived calories, respectively). These experimental diets provided sufficient amounts of protein, vitamins, and minerals according to the Nutrient Requirements of Laboratory Animals. High-fat diets components have been previously described 10. All animals had free access to water and chow (40 g/day). After starting the experimental protocol, body weight (BW) was recorded weekly.

The experimental protocol was divided into three periods: induction (3 weeks), exposure to obesity (13 weeks), and resistance training protocol (10 weeks). At the end of the protocol of exposure to obesity (13 weeks of obesity initial moment), rats were submitted to composition and redistribution of groups. For constitution of two homogeneous groups, ensuring that C group was composed only of animals with characteristics of control animals and Ob group was composed only of animals with characteristics of obese animals, a confidence interval of 95% (CI) was created based on the BW means of control and obese rats. A separation point (SP) was applied between groups; a BW medium point between C upper limit and Ob lower limit. Based on this point, animals with BW above the SP were excluded from C group, and animals with BW below the SP were excluded from Ob group, as previously described 5. Thus, 25 animals from C group (C; n=25) and 21 animals from Ob group (Ob; n=21) remained in the study.

After the composition of groups, animals were redistributed into two more groups as absence or presence of resistance training (RT). Therefore, in the second stage of experimental protocol, this study was composed of four groups: sedentary control (C; n=10), control submitted to resistance training (RT; n=11), sedentary obese (Ob; n=9), and obese submitted to resistance training (ObRT; n=11).

It's important to highlight that 5 animals died from undetermined causes throughout the experimental protocol.

### Resistance Training (RT) Protocol

#### Familiarization

The RT protocol was adapted from Hornberger & Farrar 33, which consisted of climbing a vertical ladder (110 cm high, 18 cm wide, with 2 cm-grid steps, 80◦ incline) with a load apparatus affixed to the base of the rat's tail by means of plastic insulation tape. Resting chamber (20 x 20 x 20 cm) was placed on the top of the ladder, which served as a shelter during rest between climbing sets 33. The weight attached to the base of the tail was gradually increased with exercise progression. The sets were characterized from voluntarily ladder climbs of rats to the top of the ladder. Familiarization of RT and ObRT groups with the load apparatus was gradual by climbing for 3 non-consecutive days without any weight prior the RT protocol. In the familiarization period, the animals were stimulated to perform four complete climbs on the ladder, with a 60-second-rest interval between climbs 33.

#### Maximum load carrying test (MLCT)

Three MLCTs were performed during the experimental protocol (*after the familiarization period*, *before starting the experimental protocol, and after 10 weeks of RT protocol)*. The initial load was 50% of BW (first climb), and for each series completed, 30g was added to the load. The test was conducted until the animal was unable to climb the ladder. The interval between each series was 120 seconds for all tests. The highest loaded load (HLL) successfully throughout the ladder was considered the maximum load (ML), which was used for the prescription of the RT protocol intensities. In the second and third MLCTs, the load of the last training session for the beginning of the test (50% ML) was used. The following parameters were analyzed: absolute (g) and relative loads (%), and for comparison of the results obtained between MLCTs, the delta (Δ) of force was calculated using the formula: final MLCT- initial MLCT multiplied by 100/initial MLCT and expressed as a percentage (%) 33.

#### Resistance training period

Training sessions consisted of one set of climbing, using progressive loads, interspaced with 60-second intervals. The animals had to perform 4-5 climbs to the top of the ladder. The load was progressively increased from 50% of the maximum load in the first series, through 75%, 90%, until 100% of the maximum load in the fourth series. After that, if the animal completed the fourth series, it was submitted to a fifth series with 100% of the maximum load plus 30g to failure. Failure was determined when the rat could not progress up the ladder after three successive gentle stimuli to the tail. Resistance training was performed in the afternoon, for 3 times a week, from Monday to Friday for 10 weeks.

### Comorbidities Associated with Obesity

#### Blood pressure measurements

After the end of the experimental protocol, systolic blood pressure (SBP) and diastolic blood pressure (DBP) were assessed indirectly by the non-invasive tail-cuff plethysmography (Insight Equipment, Ribeirão Preto, SP, Brazil). The animals were housed in a heated chamber, with average temperature of 37ºC for 15 minutes 34. After this period, a rubber cuff with pressure transducer from 0 to 300 mmHg was connected to the proximal tail. The values of SBP and DBP were obtained through the transducer signals coupled to the computer and analyzed on software (Software Flow Pressure Meter, Insight Equipment, Ribeirão Preto, SP, Brazil). The average of three pressure readings was recorded for each animal. In addition, the mean arterial pressure (MAP) was obtained by the following formula: MAP = (SBP + 2 × DBP)/3 35.

### Glucose tolerance test (GTT)

After the end of the experimental protocol, all rats were fasted for 4-6 h prior to the glucose tolerance test. After fasting, a blood sample was collected from the tip of the tail. The basal blood glucose level of each animal was immediately determined using a handheld glucometer (Accuchek Advantage; Roche Diagnostics, Indianapolis, IN, USA). Subsequently, an injection of glucose solution (2 g/kg body wt) dissolved in water was administered intraperitoneally, and blood glucose levels were measured after 15, 30, 60, 90, and 120 min 36. Glucose intolerance was evaluated by the area under the curve (AUC) for glucose.

### Euthanasia and Collection of Biological Material

At the end of the experimental protocol, the animals were fasted for 12 to 15 hours and received an injection with sodium heparin (1000U/kg/i.p; Heparamax-s, Blau Pharmaceutic S.A., São Paulo, Brazil) for blood anticoagulation. After 30 minutes, rats were anesthetized with ketamine (50 mg/kg/ip, Dopalen, Sespo Indústria e Comércio Ltda - Vetbrands Division, Jacareí, São Paulo, Brazil) and xylazine (10 mg/kg / ip; Anasedan, Sespo Indústria e Comércio Ltda - Vetbrands Division, Jacareí, São Paulo, Brazil), and euthanized. After median thoracotomy, the heart, ventricles, fat pads of adipose tissue and tibia were separated, dissected, weighed, and measured.

### Determination of Obesity

After 26 weeks, a criterion based on the adiposity index was used to determine obesity 37,38. Body weight was measured weekly and body fat (BF) amount was determined from the dissection of the epididymal, retroperitoneal, and visceral fat deposits. The adiposity index (AI) was calculated using the following formula: AI = (total BF/final body weight) × 100 39.

### Lipid and hormonal profile

Blood samples were collected from the abdominal artery and centrifuged at 5000 rpm for 10 minutes (Eppendorf Centrifuge 5804-R, Hamburg, Germany) in dry tubes, and the plasma was collected and stored in Eppendorf tubes in the freezer at -80°C (Thermo Fisher Scientific LLC, Asheville, NC, USA). The tissues used for further analysis were collected and frozen immediately with liquid nitrogen and then packaged and stored in the freezer at -80ºC (ColdLab Ultra Freezer CL374-86V, Piracicaba, São Paulo, Brazil). Plasma was evaluated by levels of total cholesterol (T-Chol) and high-density cholesterol (HDL), and hormones (insulin and leptin). T-Chol and HDL were measured with an automatic enzymatic analyzer system (Biochemical analyzer BS-200, Mindray, China). Leptin levels were determined with the enzyme-linked immunosorbent assay (ELISA) method using commercial kits (Linco Research Inc., St. Louis, MO, USA).

### Muscle morphometric

At the end of the experimental period, animals previously anesthetized had their soleus and tibialis muscles from the left hindlimb were removed, and then weighed.

### Cardiac remodeling

Cardiac remodeling was measured by *post-mortem* morphological analysis, histological study, isolated cardiomyocyte contractile function, as well as by intracellular Ca^2+^-cycling protein analysis by Western blotting.

### Postmortem Morphological Analysis

Cardiac remodeling at the macroscopic level, which identifies presence or absence of cardiac hypertrophy, was determined by analyzing the following parameters: heart and left ventricle (LV) weights, heart and LV normalized by tibia length.

### Histological Study

LV fragments were placed in 4% paraformaldehyde solution pH 7.4, transferred to 70% ethanol solution and embedded in paraffin. Thick sections of 6 mm thickness were cut from tissue block and stained with hematoxylin-eosin solution. After HE staining was developed, slides were mounted and visualized under microscopy (40×; AX70, Olympus Optical CO, Hamburg, Germany) to determine the myocyte cross-sectional area (CSA), which was determined for at least 50 myocytes per slide with rounded shape and nucleus visible at the center of the cell 40. CSA (μm^2^) was used as an indicator of cell size, characterizing presence or absence of cardiac hypertrophy.

LV interstitial collagen fraction (%) was determined for the entire picrosirius red stained cardiac section. Further analysis of the quantification of the interstitial collagen fraction was performed using 30 to 40 fields per fragment. The histological sections were enlarged 40 times with the aid of a biological microscope (BEL Photonics Research Bio 3, Porto Alegre, Rio Grande do Sul, Brazil). The components of the cardiac tissue were identified according to color level as follows: red for collagen fibers, yellow for myocytes, and white for interstitial space. Perivascular collagen was excluded from this analysis. The analyses were performed through software (Image Pro-plus, Media Cybernetics, Silver Spring, Maryland, USA).

### Cardiomyocyte preparation

Under anesthesia, rats from each group were euthanized and the hearts were quickly removed by median thoracotomy and enzymatically isolated as previously described 41. Briefly, the hearts were cannulated and retrograde perfusion of the aorta was performed in Langendorff system (37^o^C) with a modified isolation digestion buffer solution (DB), a calcium-free solution containing 0.1 mM ethylene glycol-bis (ß-aminoethyl ether)-N, N, N', N'-tetraacetic acid (EGTA) and N-[2-hydro-ethyl]-piperazine-N'-[2-ethanesulfonic acid] (HEPES) equilibrated with 5% CO_2_-95% O_2_ for ~3 to 5 min. The composition of DB solution was (mM): 130 NaCl, 1.4 MgCl_2_, 5.4 KCl, 25 HEPES, 22 glucose, 0.33 NAH_2_PO4, and pH 7.39. Afterwards, the hearts were perfused for 15-20 minutes with a DB solution containing 1 mg/ml collagenase type II (Worthington Biochemical Corporation, UK) and Ca^2+^ (1 mM). The digested hearts were then removed from the cannula, cut down and placed into small conical flasks with DB solution containing collagenase supplemented with 0.1% bovine serum albumin and Ca^2+^ (1 mM). After that, this process was performed 2 more times without collagenase, with addition of 1.6 and 3.12 µL (1.0 mM CaCl_2;_ stock solution). Each stage containing cells and solutions was held for approximately 10 minutes. Then, the supernatant was removed, and the myocytes were resuspended in Tyrode's buffer containing (in mM): 140 NaCl, 10 HEPES, 0.33 NaH_2_PO_4_; 1 MgCl_2_, 5 KCl, 1.8 CaCl_2_, 10 glucose. Only calcium-tolerant, quiescent, rod-shaped cardiomyocytes showing clear cross-striations were studied. The isolated cardiomyocytes were used within 2-3 h of isolation.

### Cardiomyocyte contractility

Briefly, isolated cells were placed in an experimental chamber with a glass coverslip base mounted on the stage of an inverted microscope (IonOptix, Milton, MA, USA) edge detection system with a 40× objective lens (Nikon Eclipse - TS100, USA). Cells were immersed in Tyrode's solution containing 1.8 mM CaCl_2_ and field stimulated at 1 Hz (20 V, 5ms duration square pulses). Cell shortening in response to electrical stimulation was measured with a video-edge detection system at a 240-Hz frame rate (Ionwizard, Ion Optix, Milton, MA, USA), and the contractile parameters were evaluated. Fractional shortening (expressed as a percentage of resting cell length), maximal rate of contraction and relaxation, times to 50% contraction, and relaxation were measured. The total numbers of cells analyzed are described in the legend of each figure.

### Western Blot

Protein expression of sarcoplasmic reticulum Ca^2+^-ATPase (SERCA2a), phospholamban (PLB), PLB serine^16^ phosphorylation (pPLB ser^16^) and β-actin were determined by Western blot analysis. SERCA2a/PLB and pPLBser^16^/PLB ratios were also determined. Briefly, LV samples from C (n= 5), Ob (n= 4), RT (n= 4), and ObRT (n= 5) rats were frozen in liquid nitrogen and homogenized in a buffer containing 50 mM potassium phosphate buffer (pH 7.0), 0.3 M sucrose, 0.5 mM dithiothreitol (DTT), 1 mM EDTA (pH 8.0), 0.3 mM phenylmethylsulfonyl fluoride (PMSF), 10 mM sodium fluoride (NaF), and phosphatase inhibitor cocktail (1:100; Sigma-Aldrich, St. Louis, MO, USA). The samples were subjected to SDS-PAGE in 12-15% polyacrylamide gels depending on the molecular weight of the protein. After electrophoresis, the separated proteins were transferred to nitrocellulose membranes (Bio-Rad Biosciences, USA), using the Mini Trans-Blot system (Bio-Rad, Hercules, CA, USA), and containing transfer buffer (25 mM Tris base; 190 mM glycine; 20% methanol and 10% SDS). Equal loading of the samples (50 mg) and transfer efficiencies were monitored with 0.5% Ponceau S staining of the membrane. The blotted membrane was blocked (5% non-fat dry milk, 10 mM Tris-HCl (pH 7.5R), 100mM, NaCl, and 100% Tween 20) for 2h at room temperature and then incubated overnight at 4ºC with specific primary antibodies PLB Monoclonal Antibody, mouse IgG (ThermoFisher Scientific, USA), PLBser16 Antibody, rabbit IgG (Badrilla, UK), SERCA2a, Polyclonal Antibody, goat (Santa Cruz Biotechonology, USA). Binding of the primary antibody was detected with peroxidase-conjugated secondary antibodies (rabbit, mouse or goat IgG-HRP (Santa Cruz Biotechonology, USA) for 1.5 hours at room temperature. Protein bands were visualized via chemiluminescent detection (ECL Prime Western Blotting Detection, GE Healthcare Life Sciences, USA) in a western blot detection imaging system (FX PRO, Bruker BioSpin Corporation, USA), and quantified by densitometry using Image J Analysis software. Targeted bands were normalized to the expression of cardiac β-actin (Cell Signaling Technology, USA).

### Statistical analysis

The results are reported as means ± standard deviation (SD) and submitted to the Kolmogorov-Smirnov test. As all data presented adherence to normality, two-way analysis of variance (ANOVA) followed by Tukey's or Bonferroni *post hoc* tests were performed using Prism 8.0 software (GraphPad Prism version 8.01 for Windows; GraphPad Software, San Diego, CA, USA). The level of significance was 5%.

## Results

**Figure 1:**
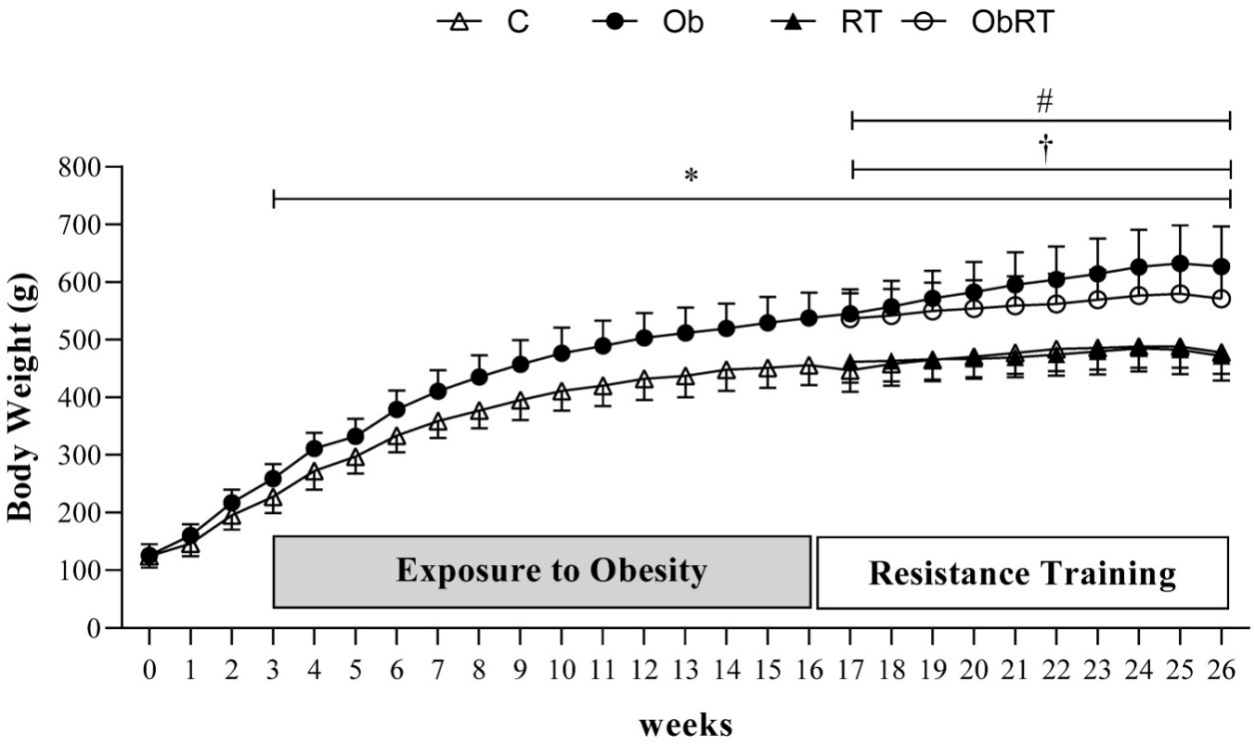
Evolution of body weight during 26 weeks of experimental protocol. Sedentary control group (C, n = 10), sedentary obese (Ob, n = 9), control submitted to resistance training (RT, n = 11) and obese submitted to RT (ObRT, n = 11). Data presented as the mean ± SD. Two-way ANOVA for repeated measurements followed by Bonferroni *post hoc* test. p<0.05 *C *vs.* Ob; ^†^RT vs. Ob; ^#^RT *vs.* ObRT.

Figure 1 shows the evolution of body weight during 26 weeks of experimental protocol. The BW was similar in the first three weeks of treatment between groups; however, Ob group presented higher BW than C in the 3^rd^ week, considered the initial moment of obesity. From 3^rd^ to 16^th^ week, there was statistical difference in BW between groups (C *vs*. Ob), characterizing the period of exposure to obesity. After that, C and Ob rats were redistributed into two more groups as absence or presence of resistance training (RT). Thus, Ob and ObRT groups presented an elevation of BW when compared with the respective control groups (C and RT), which remained significantly greater during the experimental period (17^th^ to 26^th^ week); however, the resistance training was not be able to reduce the body weight in ObRT group when compared to Ob rats. In addition, RT also promoted reduced BW in relation to Ob group (Ob>RT) from the 17^th^ to 26^th^ week.

After 26 weeks, Ob rats demonstrated a final body weight 31% and 33% greater than C and RT, respectively (Table 1). In addition, obesity promoted a substantial elevation of epididymal, retroperitoneal and visceral fat pads compared to C and RT groups, respectively. Specifically, these rats showed elevation of 125% and 128% in body fat, respectively, when compared with C and RT rats. Adiposity index was also significantly greater in this group (69%) than in C and RT rats. Nevertheless, considering the Ob and ObRT groups, the resistance training led to a significant reduction of epididymal fat (28.8%) and visceral fat pads (26.9%) when compared to Ob rats, but it was not able to reduce FBW, retroperitoneal fat pad, body fat (BF), and adiposity index (AI). In addition, ObRT group showed differences in FBW, epididymal, retroperitoneal and visceral fat pads in relation to RT (ObRT > RT), as well as for BF and AI, demonstrating obesity effect (Table 1).

**Figure 2:**
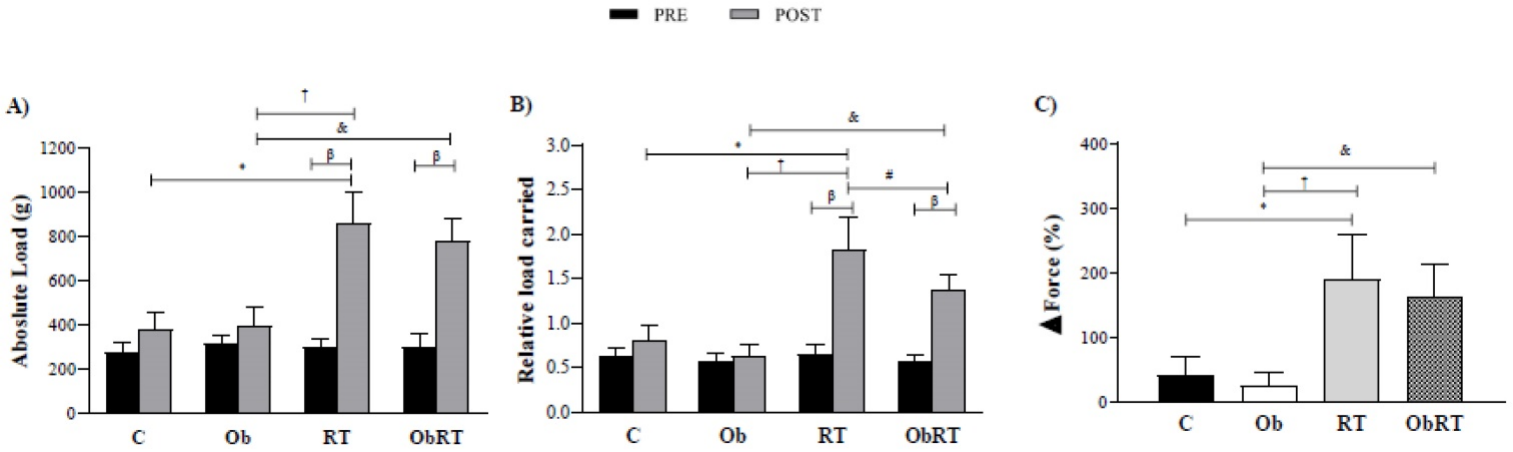
Maximum load carrying test (MLCT) data before (PRE) and after (POST) 26 weeks of experimental protocol. A) Absolute load (g); B) relative load carried (Absolute load/FBW) and C) Delta ∆ force training (%). Sedentary control group (C, n = 10), sedentary obese (Ob, n = 9), control submitted to resistance training (RT, n = 11) and obese submitted to RT (ObRT, n = 11). Data presented as the mean ± SD. Two-way ANOVA complemented with the *post hoc* Tukey test. p<0.05. *C vs. Ob; ^†^RT vs. Ob; ^#^RT vs. ObRT; ^&^Ob vs. ObRT; ^β^pre *vs.* post.

There was no statistically significant difference in absolute and relative training loads among groups in the initial MLCT (*pre training*), but the maximum workload capacity of the resistance training groups (RT *vs.* C and ObRT *vs.* Ob) was increased throughout the training period (Figure 2). Experimental groups, RT and ObRT increased significantly their maximum carrying load capacity (125% and 98%) compared with C and Ob, respectively (Figure 2A). RT also promoted elevation of absolute load when compared with Ob. In relation to relative load carried, RT and ObRT groups presented higher values ​​than sedentary groups (C and Ob). In addition, RT group had increased relative load when compared with ObRT and Ob rats (Figure 2B). Furthermore, the ∆Force was elevated in resistance training groups (RT and ObRT) when compared with the respective control groups (Figure 2C). There was no difference between RT and ObRT.

The comorbidities associated with obesity are summarized in Table 2. Obesity did not promote significant metabolic and hemodynamic alterations, but this condition only caused elevation in leptin levels (Ob > C). Nevertheless, the resistance training was able to promote a reduction in the leptin levels in ObRT in relation to Ob group (42.1%). In addition, AUC was elevated in Ob group, but there was no significant difference when compared with C (p = 0.07). However, RT was effective in the reduction of glucose and leptin levels (RT < ObRT and Ob). The other parameters, including AUC, SBP, DBP, T-Chol, and HDL, were similar among groups.

Soleus and tibialis muscles weights of animals from ObRT group were similar when compared to the RT and Ob groups, respectively. In addition, no differences were found between the ObRT and RT groups for the tibia length, as well as the soleus and tibialis normalized muscle weight (Table 3). These results were also observed in the comparison between the Ob and ObRT groups.

**Figure 3:**
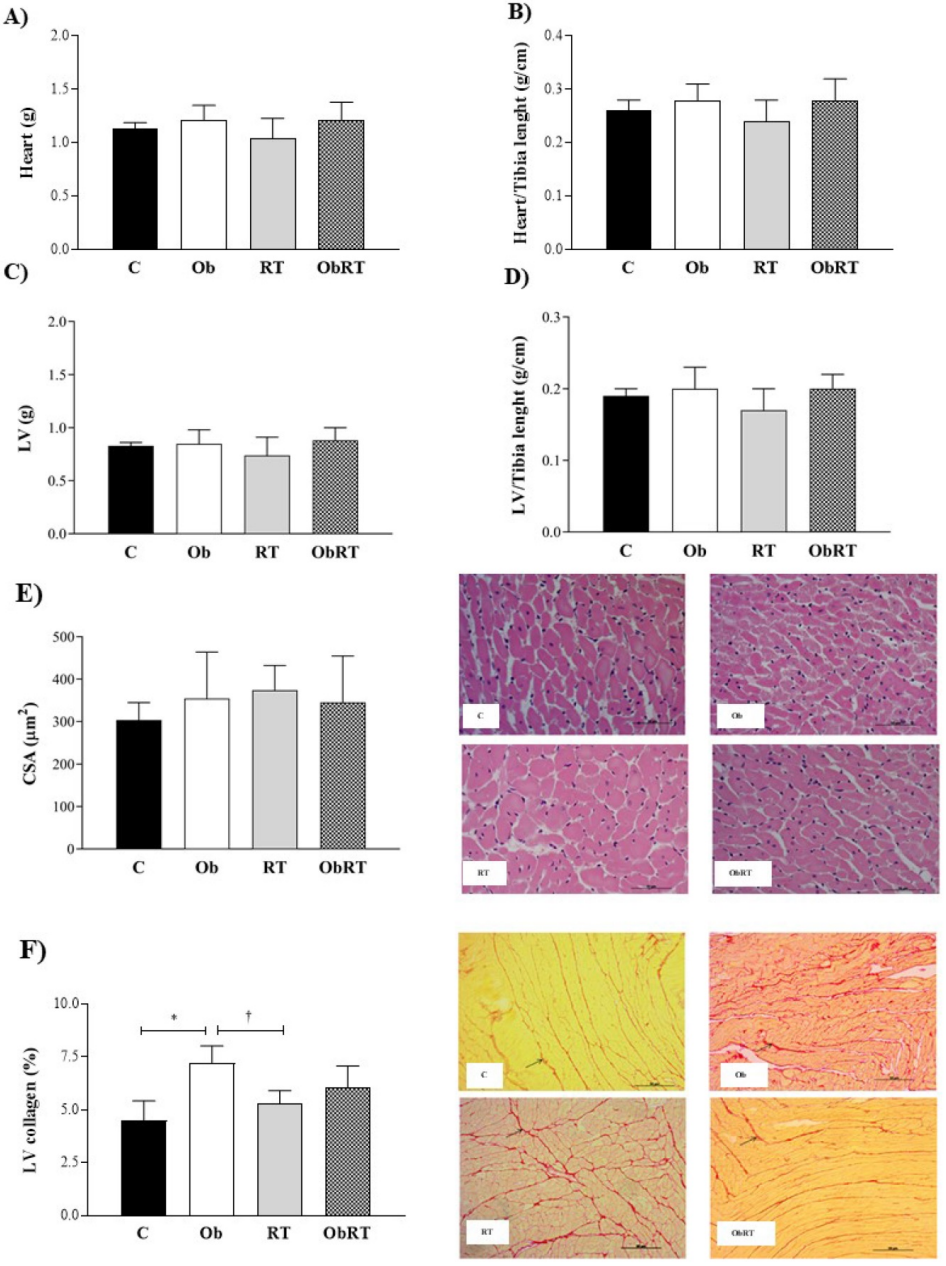
Morphological *post mortem* and histological studies performed after 26 weeks of experimental protocol. 5 animals *per group*. A) Heart weight; B) Heart normalized by tibia length; C) left ventricle (LV); D) LV normalized by tibia length; E) cross sectional area (CSA) obtained by HE staining for reticulum, and F) interstitial collagen of myocardium (40x magnification); representative *picrosirius red-stained* left ventricle (LV) section. Arrows: interstitial collagen. Sedentary control group (C), sedentary obese (Ob), control submitted to resistance training (RT) and obese submitted to RT (ObRT). Data presented as the mean ± SD. Two-way ANOVA complemented with the *post hoc Tukey's* test. p<0.05. *C *vs.* Ob; ^†^RT *vs.* Ob.

There were no differences in heart, LV, heart/Tibia, LV/Tibia and CSA among groups (Figure 3A-E). Histological analysis from LV samples revealed that interstitial collagen fraction (%) was elevated in Ob group when compared with C and RT rats (Figure 3F). However, RT was not able to reduce or prevent increase of LV collagen in ObRT, since there was no statistical difference (p = 0.25) between Ob and ObRT groups (Figure 3F).

**Figure 4:**
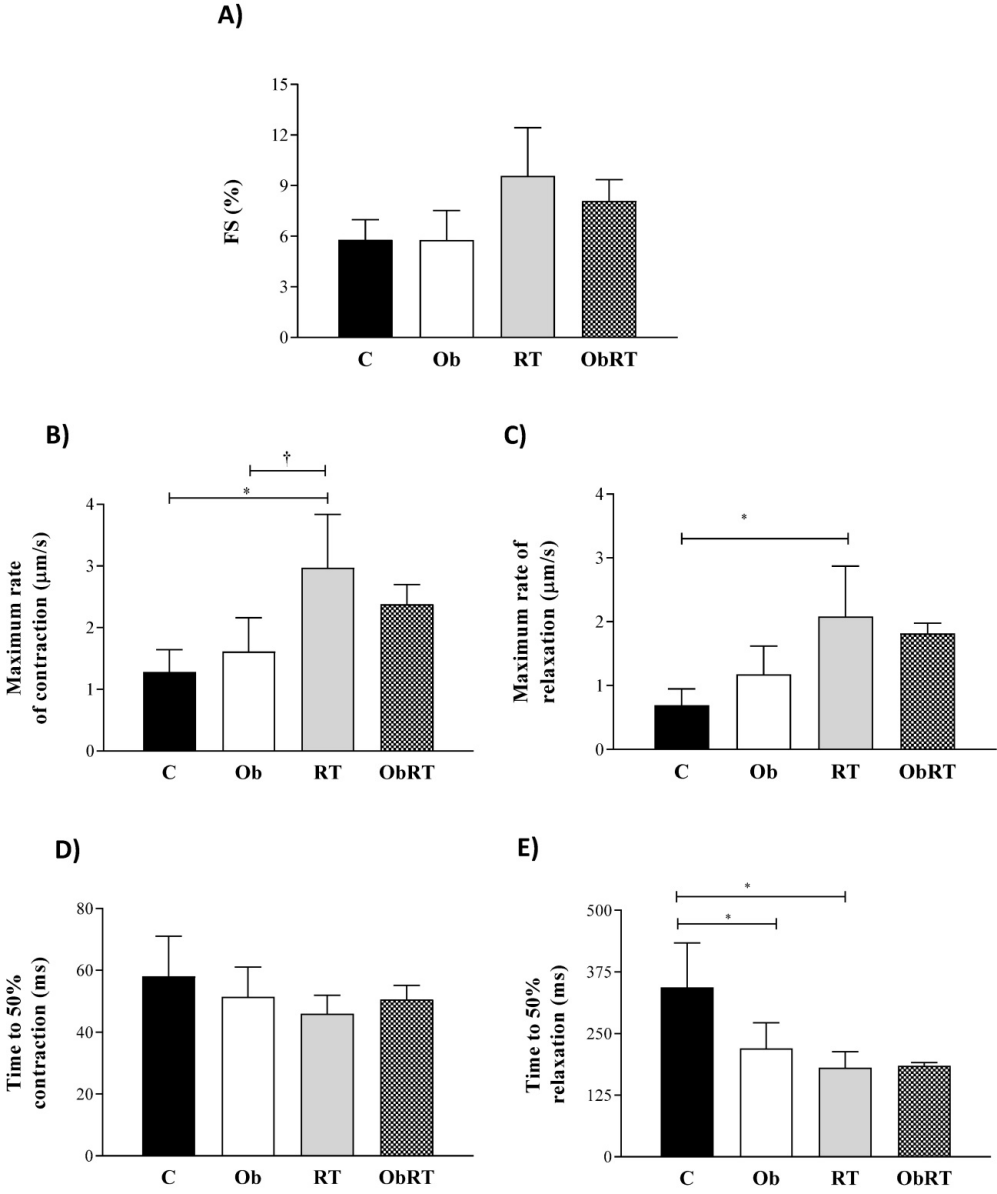
Data are presented as the mean ± SD. Four (4) animals per group. Contractile cardiomyocytes from sedentary control rats (C; cells = 11), sedentary obese (Ob; cells = 34), control submitted to resistance training (RT; cells = 29) and obese submitted to RT (ObRT; cells = 41). A) Fractional shortening (FS) expressed as % of resting cell length. B) Maximum rate of contraction. C) Maximum rate of relaxation. D) Time to 50% contraction. E) Time to 50% relaxation. Two-way ANOVA followed by post hoc Tukey's test. p<0.05. * vs. C; †RT vs. Ob.

Figure 4 summarizes the mechanical properties of isolated cardiomyocytes from all groups. RT induced a marked adaptive response in cardiomyocyte contractile function visualized by elevation in maximum rates of contraction and reduction of time to 50% relaxation in relation to C and Ob groups (Figures 4B and E). In addition, the RT promoted an elevation in maximum rates of relaxation when compared to C rats (Figure 4C). However, there was no difference in the fractional shortening among the groups, respectively (Figure 4A); fractional shortening was elevated by 65% in RT group in relation to C and Ob groups, but no differences were observed (p = 0.06). The time to 50% relaxation was reduced in Ob and RT when compared with C rats, respectively (Figure 4D), resulting in improvement of cardiomyocyte relaxation. Moreover, no differences were observed for the time to 50% contraction among groups (Figure 4D). In addition, RT was not able to improve cardiomyocyte contractile function in ObRT, since there was no statistical difference between Ob and ObRT groups (Figure 4A-E).

**Figure 5:**
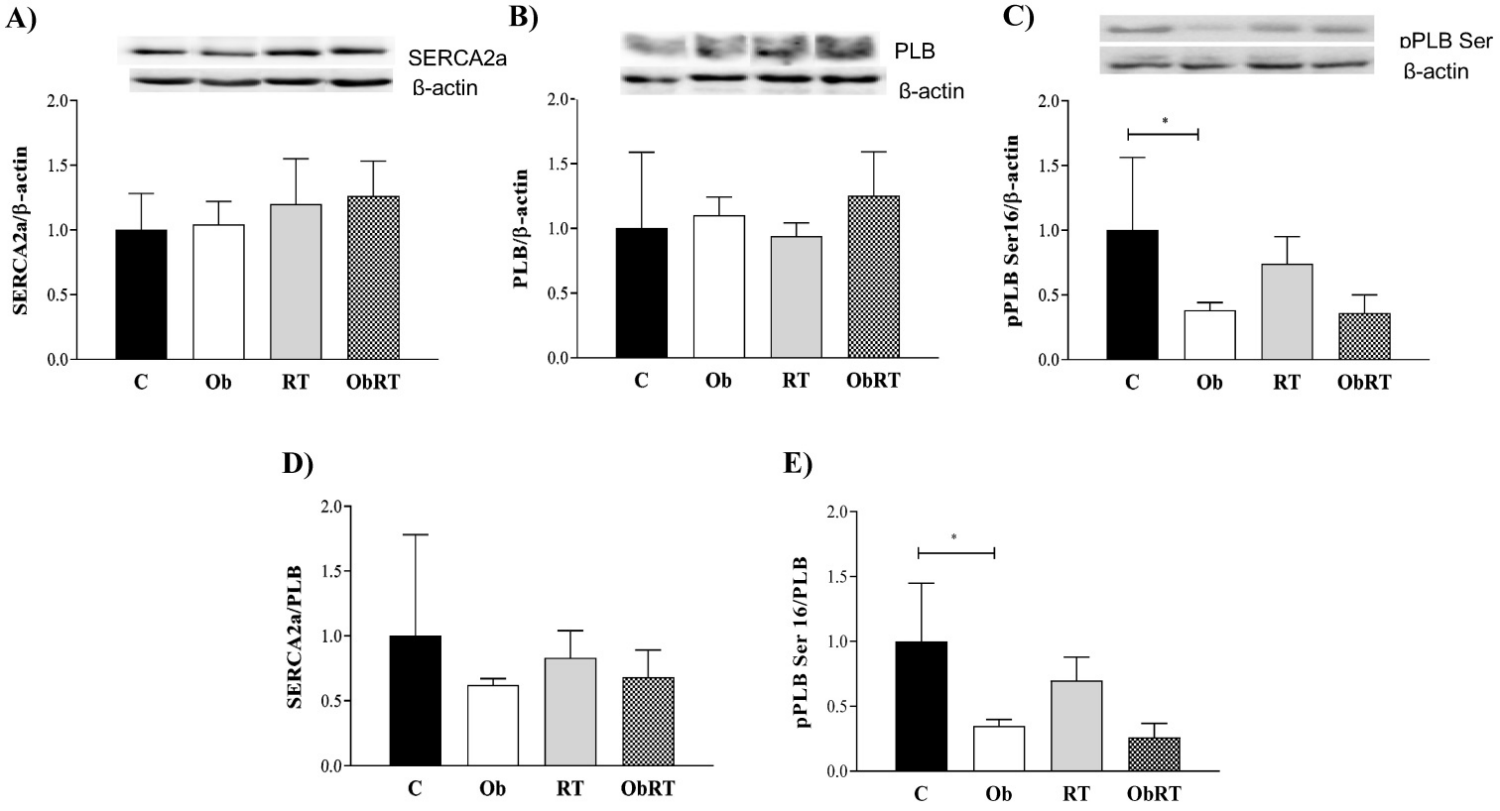
Cardiac protein expression of intracellular Ca2+ handling after 26 weeks of experimental protocol. A) Sarcoplasmic reticulum (SR) Ca2+-ATPase (SERCA2a); B) phospholamban (PLB); C) PLB serine-16 phosphorylation (pPLB Ser16); D) SERCA2a/PLB; E) pPLB Ser16/PLB. Sedentary control group (C, n = 5), sedentary obese (Ob, n = 4), control submitted to resistance training (RT, n = 4) and obese submitted to RT (ObRT, n = 5). Data presented as the mean ± SD. Two-way ANOVA complemented with the post hoc Tukey's test. p<0.05. * vs. C.

The levels of intracellular Ca^2+^ cycling proteins SERCA2a, PLB and pPLB Ser^16^ were assessed to determine the mechanism for obesity-induced changes in cardiac function and the role of RT in this process (Figure 5). There was no difference in SERCA2a, PLB and SERCA2a/PLB ratio among groups (Figure 5A, B and D). Figure 5C shows that obesity only promoted change in pPLB Ser^16^ (C: 1.00 ± 0.56 *vs.* Ob: 0.38 ± 0.06; p <0.05) when compared with C rats. Additionally, pPLB Ser^16^/PLB ratio (C: 1.00 ± 0.45 vs. Ob: 0.35 ± 0.05; p<0.05) was significantly diminished in Ob rats. Nevertheless, the resistance training was not able to reverse these alterations associated with obesity (Ob *vs.* ObRT).

## Discussion

The main findings of this study indicate that obesity promotes elevation of adiposity and hyperleptinemia, and RT was able to promote strength increase, reducing leptin levels and epididymal and visceral fat pads without changes in total adiposity and other comorbidities. Obesity caused increased deposit of myocardial collagen and reduced expression of pPLBser^16^, as well as of pPLBser^16^/PLB ratio, without contractile cardiomyocyte impairment. Surprisingly, RT was not able to reverse this damage.

The development of obesity was confirmed by higher body weight in Ob rats when compared with group C during the 3^rd^ week of experimental protocol, reiterating the efficiency of high-fat diets to induce obesity in animal models 42-44. In the current study, RT was not able to modulate the body weight in ObRT in comparison with Ob group from 17^th^ to 2^6th^ weeks. These findings are in disagreement with the studies found in the literature, indicating that RT was a viable non-pharmacological strategy to reduce body weight 26,31,44. On the other hand, with regard to the effect of RT associated with the high-fat diet, it was possible to verify the effectiveness of this type of training to reduce epididymal (40.5%) and visceral (37%) fat pads, but without changes in total adiposity. Our findings are in agreement with Speretta et al. 45, who observed that resistance training was able to decrease the area of ​​visceral and epididymal adipocytes. In addition, Leite et al. 16 showed that RT, performed 3 times a week for 12 weeks, was able to reduce fat percentage and increase fat-free mass, determining factors of body composition.

In contrast, the retroperitoneal fat deposit did not change with RT. The literature reports that body fat depots present distinct metabolic behavior, evidenced by lower expression of β3 adrenergic receptors in retroperitoneal fat 46. The authors emphasize that different body fat deposits may be more or less susceptible to lipolysis conditions according to the expression of β3 adrenergic receptors. Moreover, as explained by Tchernof et al. 47, visceral fat is more lipolytic than subcutaneous fat, considering the higher number of β-adrenergic receptors in this region. Another explanation for adiposity reduction induced by exercise may be related to its capacity to modulate mechanisms such as daily energy expenditure, resting metabolic rate and post-exercise oxygen consumption, contributing to body weight loss 48.

The literature has demonstrated that resistance training induces higher protein synthesis and muscle hypertrophy in several models 49,50. In the current study, RT and ObRT increased the absolute and relative load and strength (RT> C; ObRT> Ob), but they did not be able to promote elevation in soleus and tibialis muscles (*skeletal muscle mass*). However, the relative load in the MCLT remained lower in ObRT compared with RT rats (ObRT < RT). Interestingly, this finding point out that the condition of obesity triggers reduced functionality, since despite the greater body weight in ObRT group, the capacity to produce strength was not higher than the RT group, possibly due to the large adiposity in ObRT. Miller et al. 51 highlighted that changes in body composition influence the physical functionality, which can result in reduced functionality and physical disability. Obesity, evidenced by the excessive accumulation of adipose tissue, is a condition that favors the development of less functional capacity 52-54, corroborating the findings with the current study. Bollinger ratified that absolute strength seems to be greater in obesity, however when the strength is normalized by body weight, the relative muscle strength is reduced 55. In addition, other studies have demonstrated that in the condition of obesity, the normalization of the absolute load by the body weight in dynamic conditions, such as stair training, represents a relevant indicator of functional performance 56,57. Nevertheless, the high-intensity RT sessions enable the strength development and gain in both groups (RT and ObRT). The high-intensity RT sessions enable the strength development and gain. Recent studies have shown that this type of training increases the rat's muscular strength 45,58-61. Schoenfeld suggests that regardless of the magnitude of the load (low or high), muscle hypertrophy is achieved if training is done until voluntary muscle failure 62, but in experimental models, it is difficult to determine whether rats climb the ladder until failure. Adaptations to training are related to the amount of work performed during the exercise sessions 60. In this sense, the physical exercise induces momentary adjustments in physiology, biology, and cellular biochemistry 56, and muscle strength gain may occur through neural and/or structural adaptations 64.

Obesity has been associated with several comorbidities, such as glucose intolerance, hyperinsulinemia, insulin resistance, dyslipidemia, and hypertension 65,66. Obesity model presented only hyperleptinemia but without elevations in fasting glucose, total cholesterol and cardiovascular parameters, as well as reduction in HDL levels. Moreover, RT in obese animals did not cause changes in blood pressure, but it was able to significantly decrease plasma leptin levels compared with their respective control group. According to literature, the physical exercise is widely accepted to prevent or treat metabolic diseases, promoting different beneficial effects in obese patients and rats 16,45,67,68, but these alterations were not observed by RT used in this study. While the aerobic exercise has a clinically significant effect on cardiorespiratory fitness and metabolic control, resistance training improves insulin sensitivity and glucose tolerance, whereas improving lean body mass 69. Sertié et al. 70 identified that 10 weeks of aerobic training performed 5 days a week was ineffective in altering plasma triglyceride and cholesterol levels in obese *Wistar* rats. Specifically, regarding the resistance training, Speretta et al. 71 showed that RT was be able to prevent the increase in total cholesterol in HFD rats, but it did not promote improvements in TG and HDL levels. These findings together with previous studies suggest that resistance training induces metabolic alterations in a time-dependent manner, accomplishing favorable modulation of metabolic serum levels, especially lipid profile, and thus promoting antiatherogenic effects 72. Nevertheless, it has been well documented regarding this type of exercise that increased volume rather than increased intensity has greater impact on lipid profile 73. Another explanation to absence of metabolic beneficial alterations could be related to the way they are mediated, that is, more strongly through exercise itself or exercise-induced weight loss and subsequent improvements in body composition 74. Regarding glucose intolerance, Caponi et al. 75 showed a positive correlation between exercise and GLUT 4 expression in skeletal muscle and heart, showing that physical training improves insulin sensitivity and it has beneficial effects on metabolic syndrome. Therefore, we believe that the training volume (*3 times a week, 4-5 climbs*) in the current study was low to promote greater metabolic changes.

The literature reports that physiological cardiac hypertrophy may occur through stimulation generated by physical exercise resulting in normal or supranormal ventricular function 3. On the other hand, the maladaptive remodeling can be associated with increase in left ventricle (LV) end-diastolic-pressure, pulmonary congestion, and LV hypertrophy 3,76. In addition to myocyte hypertrophy, another element involved in the cardiac remodeling process is the interstitial connective tissue. Increases in collagen content may cause myocardial dysfunction due to impairment of the ventricular compliance, as well as changes in cardiac geometry. In this study, elevation of myocardial collagen was observed in obesity condition but without LV hypertrophy. Nevertheless, resistance training was not able to promote LV hypertrophy or to reverse or prevent the collagen accumulation in Ob group.

Some researchers point out that the overload generated on the heart depends on the type of training, aerobic or anaerobic 20,77,78. In this context, in RT, there is increase in the pressure overload on the heart, which may generate physiological cardiac hypertrophy 20, 77,78. An explanation for our results may be related to absence of pressure overloads, which are evidenced by normal systolic and diastolic arterial pressures among the experimental groups. Another mechanism may be related to leptin, which partially mediates the process of cardiac hypertrophy by the mitogen-activated protein kinase (MaPK p38), which regulates various cellular processes; however, our results showed that RT promotes reduction in leptin levels in obesity condition. Thus, absence of cardiac hypertrophy in this study can be explained by the total RT volume. According to Cassilhas et al. 79, some adaptive effects are not seen with different training volumes.

As regards the cardiomyocyte contractile function, obesity did not cause impairment of cardiac function demonstrated by similar fractional shortening percentage and lower time to 50% relaxation compared with group C, but RT alone promoted improvement in maximum rate of contraction and relaxation. After exercise stimulus, heart rate and cardiac contractility increase to meet the metabolic demands of the body, which is facilitated by elevation of cardiomyocyte contraction; it is partially achieved by the increased release of stored Ca^2+^ within the sarcoplasmic reticulum (SR) binding to components of the contractile apparatus and sarcomere remodeling 80,81. However, in disagreement with our initial hypothesis, RT was not able to promote favorable adaptations to cardiovascular system in obesity condition as improvement in contractile function during excitation-contraction coupling, or to reverse the phospholamban phosphorylation regarding serine^16^ damage.

The literature has largely investigated the benefits of aerobic exercise training for cardiac function and proteins related to myocardial intracellular Ca^2+^ handling in both healthy and pathological conditions 13,21,22,82. However, the effects of RT on Ca^2+^ handling and its association with myocardial intracellular proteins have been poorly investigated. Although the literature reports that physical exercise favors Ca^2+^ transport and increased Ca^2+^ availability for cardiac contractility 83,84, in this study, RT did not modulate the protein expression of SERCA2a and PLB in obesity condition. SERCA2a and PLB proteins are highlighted by their important function in mediating Ca^2+^ recapture for sarcoplasmic reticulum 85, and PLB regulates SERCA2a activity by phosphorylation and dephosphorylation of domain sites, which are found in the transmembrane and cytosolic medium 86. Elevated SR calcium reuptake may also be caused by higher SERCA2 pumping capacity without any change in the amount of SERCA2 or PLB. The unphosphorylated form of PLB inhibits SERCA2 activity, whereas the phosphorylated form of PLB dissociates from inhibitor leading to increased pumping activity. PLB is phosphorylated at the serine residue via β-adrenergic pathway and at the threonine residue primarily via calcium/calmodulin kinase II.

In this research, only the serine phosphorylation^16^ was evaluated, reported as the main mediator of the positive effect on cardiac contractility and prevalent in relation to the threonine^17^ 87. Our results showed that obesity condition impaired pPLBser^16^, represented by lower cardiac expression of this protein in Ob group, but it was notable to affect the contractile function, since it improved relaxation. Nevertheless, RT approach was not able to reverse the pPLBser^16^ damage or to improve the cardiac function. Although we did not analyze all these possible mechanisms involved in Ca^2+^ handling, it is possible that the phospholamban phosphorylation at threonine-17 (PLB Thr^17^) was elevated in RT and this finding did not allow reversion of PLB at Ser^16^ downregulation. In this sense, an important feature of PLB phosphorylation at Ser^16^ is that this site is more physiologically important than Thr^17^ mainly due to the lower level of PLB Thr^17^ 87,88. Phosphorylation of PLB at Thr^17^ must be potentiated by Ser^16^ phosphorylation, and Thr^17^ phosphorylation has a negligible effect after Ser^16^ has been phosphorylated 14,88. Another possible explanation for the failure to prevent and/or reverse the damage to phosphorylation at Ser^16^ visualized in obese rats may be related to improvement in β-adrenergic system regulation.

## Conclusion

Resistance training represents a relevant non-pharmacological treatment in improving body composition and obesity biomarkers, expressed by reduced visceral and epididymal fat pad and plasma leptin levels. However, positive alterations in cardiomyocyte contractile and Ca^2+^ handling, as well as in reversing the collagen accumulation and phospholamban phosphorylation regarding serine^16^ damage in obesity condition were not observed.

## Figures and Tables

**Table 1:** General characteristics

Variables	Experimental Groups			
	C	Ob	RT	ObRT
FBW (g)	478 ± 37	627 ± 69*	472 ± 43^†^	571 ± 60^#^
Epididymal fat pad (g)	7.4 ± 2.0	15.6 ± 4.6*	6.9 ± 1.0^†^	11.1 ± 3.2^#&^
Retroperitoneal fat pad (g)	12.1 ± 2.6	28.2 ± 7.1*	12.7 ± 1.1^†^	24.6 ± 7.3^#^
Visceral fat pad (g)	8.2 ± 1.5	18.6 ± 5.9*	7.7 ± 0.9^†^	13.6 ± 4.6^#&^
BF (g)	27.7 ± 5.3	62.4 ± 17.1*	27.3 ± 2.1^†^	49.3 ± 14.4^#^
AI (%)	5.8 ± 0.9	9.8 ± 1.8*	5.8 ± 0.7^†^	8.5 ± 1.8^#^

Values presented as mean ± SD. n = number of animals. Sedentary control group (C, n = 10), sedentary obese (Ob, n = 9), control submitted to resistance training (RT, n = 11) and obese submitted to RT (ObRT, n = 11). FBW = final body weight; BF: body fat; IA = adiposity index. Two-way ANOVA followed by Tukey *post hoc* test. p<0.05. *C *vs.* Ob; ^†^RT *vs.* Ob; ^#^RT *vs.* ObRT; ^&^Ob *vs.* ObRT.

**Table 2:** Comorbidities associated with Obesity

Variables	Experimental Groups			
	C	Ob	RT	ObRT
Glucose (mg/dL)	109 ± 6	113 ± 8	101 ± 7^†^	106 ± 11
AUC (mg/dL/min)	220 ± 15	270 ± 55	233 ± 33	270 ± 54
SBP (mmHg)	124 ± 14	129 ± 17	129 ± 17	124 ± 18
DBP (mmHg)	95 ± 13	112 ± 21	104 ± 16	98 ± 21
T-Chol (mg/dL)	68 ± 9	62 ± 7	64 ± 10	54 ± 7
HDL (mg/dL)	24 ± 3	25 ± 2	25 ± 3	25 ± 5
Leptin (ng/mL)	6 ± 3	19 ± 5*	5 ± 2^†^	11 ± 7^#&^

Values presented as mean ± SD. 9 animals per group. Sedentary control group (C), sedentary obese (Ob), control submitted to resistance training (RT) and obese submitted to RT (ObRT). AUC: area under the curve for glucose; SBP: systolic blood pressure; DBP: diastolic blood pressure; T-Chol: total cholesterol; HDL: high-density lipoprotein. ANOVA followed by Tukey *post hoc* test. p<0.05. *C *vs.* Ob; ^†^RT *vs.* Ob; ^#^RT *vs.* ObRT; ^&^Ob *vs.* ObRT.

**Table 3:** Muscle morphometric data

Variables	Experimental Groups			
	C	Ob	RT	ObRT
Soleus Weight (g)	0.176 ± 0.028	0.192 ± 0.035	0.167 ± 0.028	0.199 ± 0.038
Tibialis Weight (g)	0.858 ± 0.108	0.956 ± 0.148	0.865 ± 0.130	1.002 ± 0.137
Tibia Length (cm)	4.34 ± 0.13	4.41 ± 0.10	4.34 ± 0.11	4.45 ± 0.04
Soleus /tibia (mg/cm)	40.52 ± 5.93	43.68 ± 8.06	38.42 ± 5.84	44.89 ± 8.81
Tibialis/tibia (mg/cm)	197.61 ± 22.07	216.67 ± 33.12	199.29 ± 26.98	225.25 ± 30.81

Values presented as mean ± SD. Sedentary control group (C; n = 10), sedentary obese (Ob; n=7), control submitted to resistance training (RT; n = 10) and obese submitted to RT (ObRT; n = 9). ANOVA followed by Tukey *post hoc* test.
